# Selective Photocatalytic Disinfection by Coupling StrepMiniSog to the Antibody Catalyzed Water Oxidation Pathway

**DOI:** 10.1371/journal.pone.0162577

**Published:** 2016-09-12

**Authors:** Elizabeth M. Wurtzler, David Wendell

**Affiliations:** Department of Biological, Chemical, and Environmental Engineering, College of Engineering and Applied Science, University of Cincinnati, Cincinnati, Ohio, United States of America; Louisiana State University Health Sciences Center, UNITED STATES

## Abstract

For several decades reactive oxygen species have been applied to water quality engineering and efficient disinfection strategies; however, these methods are limited by disinfection byproduct and catalyst-derived toxicity concerns which could be improved by selectively targeting contaminants of interest. Here we present a targeted photocatalytic system based on the fusion protein StrepMiniSOG that uses light within the visible spectrum to produce reactive oxygen species at a greater efficiency than current photosensitizers, allowing for shorter irradiation times from a fully biodegradable photocatalyst. The StrepMiniSOG photodisinfection system is unable to cross cell membranes and like other consumed proteins, can be degraded by endogenous digestive enzymes in the human gut, thereby reducing the consumption risks typically associated with other disinfection agents. We demonstrate specific, multi-log removal of *Listeria monocytogenes* from a mixed population of bacteria, establishing the StrepMiniSOG disinfection system as a valuable tool for targeted pathogen removal, while maintaining existing microbial biodiversity.

## Introduction

Disinfection strategies employing photosensitizing agents have been used in a clinical setting to treat periodontitis [1]; and extended to water treatment applications, using porphyrins [2] chlorins [3], and nanoparticles as the antimicrobial agents, with the latter being the most popular [4,5,6,7,8,9]. Semiconductor-based nanoparticles have demonstrated disinfection capability against a wide range of contaminating organisms including gram positive [5,10,11] and gram negative bacteria [5,12,13], fungi [5,14], viruses [10,15,16], and protozoa [17,18]. Despite the popularity and demonstrated efficacy of nanoparticles, an increasing body of evidence is revealing the carcinogenic and cytotoxic properties of these materials [19,20], which are readily transported and can persist in environmental water [21] negatively impacting populations of aquatic organisms [22,23,24], ultimately representing a danger to the very water they disinfect.

Due to the low concentration of many aquatic biohazards and the cytotoxicity of many nanoparticles and other photosensitizers, creation of a new selective oxidant is desirable to minimize competition from other sources, including natural organic matter. Targeting the photosensitizer results in a 10-fold reduction in energy usage to achieve the same levels of microbial removal [25] and targeted photosensitizers can decrease the amount of photosensitive material required by up to 200 times the amount required for effective removal using the photosensitizer alone [26]. Targeted disinfection began before the more broad spectrum antibiotic era, beginning with bacteria specific phage therapies to treat bacterial infections [27,28,29]. More recently, bacteriophage applications have also been used to remove pathogenic bacteria from water filtration systems [30], biofilms [31], and targeted photodisinfection of bacteria including *Staphylococcus aureus* [32,33]. However, phage treatments are limited by the rapid coevolution of resistant bacteria [34], which quickly render the therapeutic agent ineffectual.

Targeting strategies can also employ antibodies [35] and often increase photodynamic therapy (PDT) efficiency through improved solubility and decreased hydrophobicity [36]. These strategies have the advantage of increased oxidation potential through the catalysis of H_2_O_2_ formation from singlet oxygen (^1^O_2_) via the antibody catalyzed water oxidation pathway (ACWOP), a property that appears to be universal among antibodies given the reaction center location on a conserved region of the antigen binding fragment [37]. The inter Greek key protein domain (IGKD), a combination of two Greek key protein motifs that are unique to antibodies and T-cell receptors, serves as the postulated catalytic site for H_2_O_2_ production [38,39]. Structural analysis has found that antibody tryptophan 163, a conserved residue located on the light chain, is the center of the catalytic region, possibly due to its large solvent accessible area [40]. While the complete pathway from ^1^O_2_ to H_2_O_2_ has yet to be fully elucidated, trioxidane (H_2_O_3_) and hydrogen trioxy radicals (HO_3_) have been identified as potential reaction intermediates [38,41,42].

The catalytic H_2_O_2_ formation from ^1^O_2_ and water via a conserved inter antibody Greek key domain [37,38,40] lends itself to further investigation of disinfection potential, particularly due to the reported stability of H_2_O_2_ generation [39,42], which remains stable for extended periods of time despite exposure to its reactive oxygen product. Previous work has used specific and nonspecific antibodies in conjunction with hematoporphyrin IX as the ^1^O_2_ source to disinfect *E*. *coli* [43]. However, this effect was observed at 4°C, a temperature too low for many real world surface water disinfection applications. Other work has correlated the increased presence of antibodies or their antigen binding fragments with improved pathogen removal [44,45,46] and treatment outcomes [47]. Although antibody disinfection has been found to be more effective than antibiotics for some microorganisms [45], the full potential of antibody photocatalytic disinfection remains unexplored.

**Fig 1:**
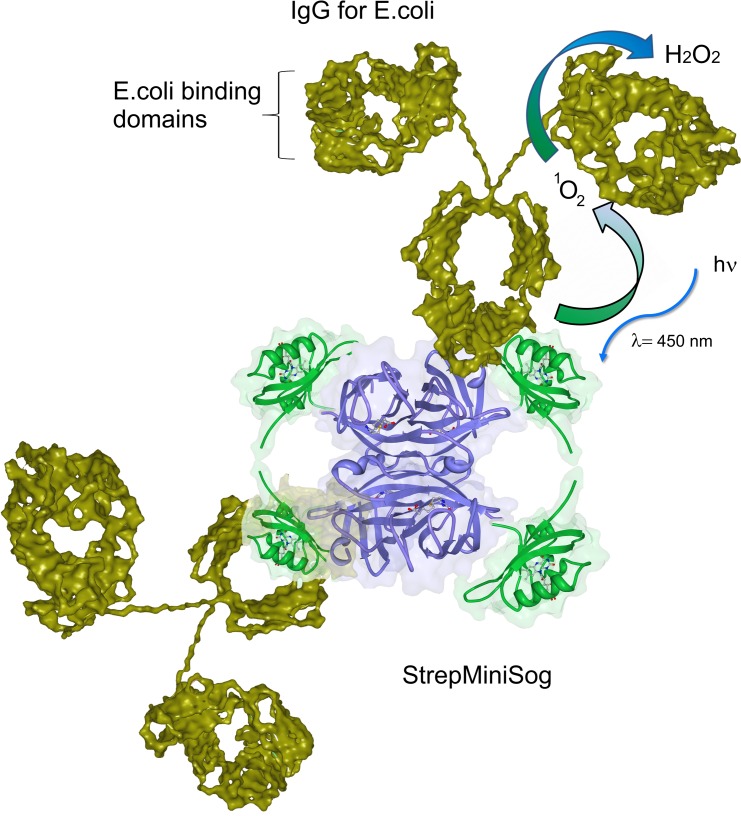
Representation of the artificial IgM created by the binding of biotinylated antibodies to SMS and its production of H_2_O_2_ by exposure to blue light. Streptavidin subunits are purple, miniSOG domains are green and IgG moieties are gold.

This study introduces a novel, targeted protein photodisinfection system based on StrepMiniSOG (SMS), a ^1^O_2_ generating fluorescent protein with biotin binding capabilities created from the Light-Oxygen-Voltage domain of *Arabidopsis thaliana* and streptavidin [48,49]. The current proteinaceous disinfection system combines the SMS fusion protein with biotinylated antibodies specific to bacteria, creating an artificial IgM (Fig 1). SMS acts as the photocatalyst, generating ^1^O_2_ from blue light, and the antibody both catalyzes and targets the H_2_O_2_ derived from ^1^O_2_ to the bacterial pathogen (Fig 1). The resulting H_2_O_2_ generation is an antibody catalyzed product originating from the ^1^O_2_ generated by SOG, but is also targeted, unlike the reactive oxygen species (ROS) generated from nanoparticles and other photocatalytic systems. Because SMS is a streptavidin-SOG conjugate, it can be universally combined with a variety of commercial biotinylated antibodies, enabling disinfection to specific pathogens of interest.

## Materials and Methods

All reagents were purchased from Sigma-Aldrich (St. Louis, MO) unless otherwise noted. The SMS gene was constructed via PCR and purified from *E*. *coli* as previously described [49]. Briefly, *E*. *coli* BL21(DE3) cells hosting an inducible SMS gene were grown for 24 hours at 25°C in Overnight Express (EMD Millipore, Billerica MA), followed by French Press lysis and purification using nickel affinity chromatography with imidazole selectively removed by dialysis using 10KMW Slide-a-lyzer cartridges (Pierce) before use. Disinfection results were analyzed according to Chick’s Law, which is described by *N* = *N_o_e*^−*kt*^, where N is the number of microorganisms remaining, N_o_ is the initial number of microorganisms, k is the disinfection constant, and t is the contact time.

### Reactive Oxygen Species Measurements

For ROS measurements, SMS was purified and analyzed for ^1^O_2_ production as described previously [49]. Sodium azide found with the commercial biotinylated antibody (Thermo Fisher, Waltham, MA) was selectively removed via dialysis using 20KMW Slide-a-lyzer cartridges (Pierce) and combined with SMS and 3,3'-diaminobenzidine (DAB) for polymerization experiments. SMS and the biotinylated antibody (SMS+Ab) were combined in a 1:2 molar ratio, with bovine serum albumin (BSA) and the antibody (BSA+Ab) at an identical ratio serving as a ^1^O_2_ free control. These mixtures were added to 0.5mg/mL DAB solution in 50mM Tris, pH 7.6 and irradiated with 450nm light (460 μmol m^-2^ s^-1^). ROS production, as measured by DAB polymerization, was quantified by monitoring absorbance at 595nm using a Flexstation 3 Plate Reader (Molecular Devices, Sunnyvale, CA). Linear DAB polymerization rates were determined using Origin (OriginLab, Northampton, MA). H_2_O_2_ generation of the SMS-antibody complex was quantified using the Amplex Red method [50]. SMS and the biotinylated *E*. *coli* antibody (EAb, Thermo Fisher Scientific Cat# PA1-73031, RRID:AB_1017147) were mixed in a 1:2 molar ratio and added to the Amplex Red reaction solution. Samples were exposed to 450nm light (460 μmol m^-2^ s^-1^) and fluorescence was measured every two minutes for 1 hour using an excitation and emission of 568nm and 570nm, respectively, with excitation cutoff set at 570nm. Fluorescent measurements were taken in the FlexStation3 Plate Reader. Control measurements were taken using BSA to replace SMS. All measurements were taken in quadruplicate.

### Disinfection Trials

*E*. *coli* used in monoculture disinfection trials were grown in LB broth to a concentration of 10^9^cells/mL. Cells were harvested by centrifugation at 3200g for 10 minutes and washed three times with 0.9% NaCl. Cells were aliquoted and stored at -80°C in 10% glycerol. For disinfection trials, cells were thawed at 37°C for 30 minutes and at least two aliquots were combined to minimize variability across aliquots. After thawing, cells were diluted to a concentration of 10^6^cells/mL in 0.9% NaCl and added to SMS and EAb or tryptophan mixed in a 1:2 molar ratio. The cells were exposed to 450nm light (460 μmol m^-2^ s^-1^) for time periods of 15 minutes, 30 minutes, 60 minutes, and 90 minutes. After exposure, the cells were spun down to remove excess SMS-EAb and stained using the Live/Dead Baclight Bacterial Viability Kit (Life Technologies). Fluorescence was measured in the FlexStation3 using an excitation of 485nm and emissions of 530nm and 630nm, with cutoffs set at 515nm and 615nm. All trials were repeated four times. For control experiments, BSA or tryptophan was used in place of EAb. Chick's Law [51] was used to determine disinfection kinetics.

To label *E*. *coli*, cells were mixed with SMS and the EAb in a 1:2 molar ratio and incubated at room temperature for 1 hour. Following incubation, cells were washed three times with sterile 0.9% NaCl. For labeling of *Listeria monocytogenes*, cells were washed with 0.25% BSA and 0.15M NaCl and Texas Red-wheat germ agglutinin was added at a final concentration of 50μg/mL. Cells were incubated at room temperature for 15 minutes and washed with 0.25% BSA and 0.15M NaCl. Imaging was performed by fluorescent microscopy at 60X magnification.

For selective disinfection trials, *L*. *monocytogenes* were grown in Brain Heart Infusion (BHI) broth and used at a concentration of 7.8x10^5^cfu/mL and *E*. *coli* were grown in LB broth and used at a concentration of 10^8^cfu/mL. Bacterial solutions were added to SMS and the biotinylated *L*. *monocytogenes* antibody (LAb, Thermo Fisher Scientific Cat# PA1-85650, RRID:AB_934475) mixed in a 1:4 molar ratio. The cells were exposed to 450nm light (460 μmol m^-2^ s^-1^) for time periods of 30 minutes, 60 minutes, and 120 minutes. After exposure, the number of cells remaining was determined by plating bacterial solutions on LB-Carbenicillin plates (*E*. *coli* counts) or BHI-Chloramphenicol plates (*L*. *monocytogenes* counts). All trials were done in triplicate. First order rates of disinfection were determined using linear regression.

## Results and Discussion

### Singlet Oxygen Based Disinfection

**Fig 2:**
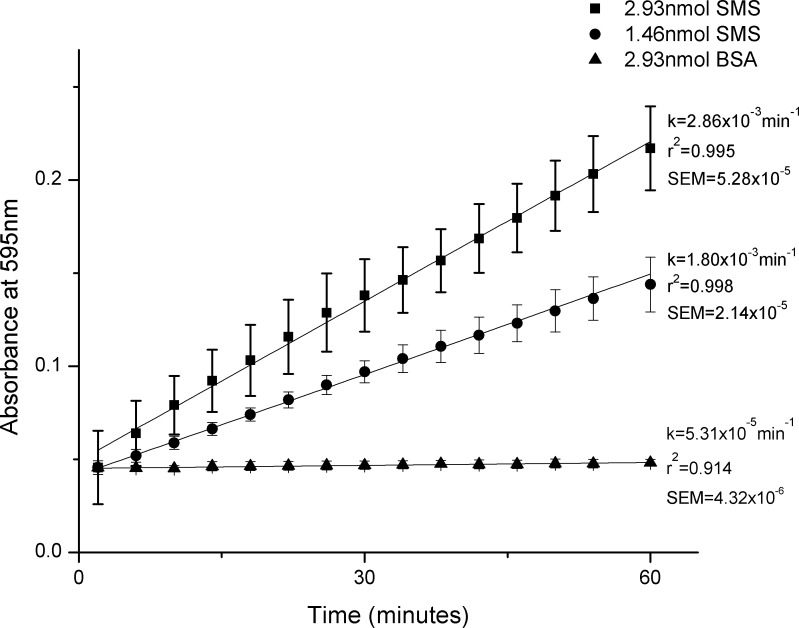
SMS Polymerization of DAB. Rates of singlet oxygen generation were found to be 2.86x10^-3^min^-1^ (r^2^ = 0.995, SEM = 5.28x10^-5^), 1.80x10^-3^min^-1^ (r^2^ = 0.998, SEM = 2.14x10^-5^), and 5.31x10^-5^min^-1^ (r^2^ = 0.910, SEM = 4.32x10^-6^) for 2.93nmol SMS, 1.46nmol SMS, and 2.93nmol BSA, respectively, as measured by increased absorbance at 595nm due to DAB polymerization. Error bars represent the standard deviation(n = 8).

While singlet oxygen is most notably used for decomposition, it has the ability to catalyze the polymerization of 3,3'-Diaminobenzidine (DAB), which is a convenient measure of singlet oxygen production via visible light absorbance [52]. As a result, direct absorbance measurements of DAB polymerization were used to determine the singlet oxygen generation rates of SMS and the BSA control [52] (Fig 2). The DAB polymerization rates for SMS were found to be 2.87x10^-3^min^-1^ and 1.96x10^-3^min^-1^, for 2.93nmol and 1.46nmol SMS, respectively. Unsurprisingly, the BSA control did not appreciably polymerize DAB.

Surprisingly, disinfection studies using only SMS showed minimal disinfection efficiency, indicating that ^1^O_2_, as it is generated from SOG is not effective at killing bacteria (S1 Fig). While efficient ^1^O_2_ production has been recorded [49] (Fig 2), the protein is much larger than other photosensitizers used to demonstrate singlet oxygen disinfection [53,54,55], indicating that extracellular production of ^1^O_2_ may not be sufficient, an observation consistent with the 2μs lifetime of ^1^O_2_ in aqueous solutions [56]. Disinfection using multimers of SOG concatenated into single polypeptide chains [49] was examined, but these proteins did not show any improved functionality over SMS (S2 Fig).

Given the unimpressive disinfection from SMS alone, a longer lived ROS was sought to improve disinfection. Previous work has suggested the formation of O_3_ from certain amino acids, including tryptophan, in the presence of ^1^O_2_ that led to removal of *E*. *coli* from solution [57], and also the reported IgA catalyzed formation of O_3_ from ^1^O_2_ [44]. Further, tryptophan residues present in antibodies have been identified as the catalytic site for H_2_O_2_ production [40], suggesting a protein mediated pathway for a longer lived ROS from ^1^O_2_ generation. As tryptophan residues present in antibodies have been suggested as the catalytic site for H_2_O_2_ production [40] and free tryptophan (Trp) has been postulated to produce ozone in the presence of ^1^O_2_ [57], tryptophan was added to the SMS disinfection trials to facilitate production of a longer lived ROS; however, the SMS+Trp combination did not provide any disinfection benefits (S3 Fig). A disinfection mixture containing SMS and PEG in equimolar amounts as Trp served as an osmotic control (S3 Fig).

Use of SMS alone or SMS augmented with free tryptophan was found to provide ineffective disinfection even at light exposure times up to 90 minutes, indicating that extracellularly generated ^1^O_2_ from SMS is unable to eliminate *E*. *coli* from solution. This result was surprising when considered in conjunction with the efficient ROS production of SMS and the production of O_3_ expected from the combination of SMS and free tryptophan [57]. However, with the exception of protein based catalysts, all ^1^O_2_ producing photosensitizers applied as PDT agents are below 1000Da in molecular weight [58] making it probable that SMS is not internalized by the cell as other photosensitizers are, leading to reduced DNA and protein oxidation by ^1^O_2_. This reinforces the notion that other photosensitizing compounds must diffuse into the cell to be effective photoinactivators, and as a result, pose a potential health hazard to downstream organisms exposed to the photosensitizer treated water [59]. Indeed, when ^1^O_2_ producing proteins such as miniSOG or Killer Red are expressed within the cytoplasmic interior of cells, they are capable of causing cell death [60,61,62], further indicating that ^1^O_2_ must be internalized to disinfect effectively. Given the short lifetime of ^1^O_2_ in aqueous solution [56], it is likely the miniSOG generated ^1^O_2_ was limited by diffusional distance and nonspecific oxidation. Therefore, it was necessary to enhance the ROS production from SMS to create a useful disinfection oxidant.

### Disinfection via the Antibody Catalyzed Water Oxidation Pathway

Due to the catalytic formation of H_2_O_2_ from ^1^O_2_ via the antibody catalyzed water oxidation pathway (ACWOP), antibodies were chosen to augment ROS production from SMS, providing a longer lived and stronger oxidant in H_2_O_2_ upon blue light irradiation of the SMS-antibody complex. To confirm increased and longer lived ROS production by ACWOP, DAB polymerization by the SMS-antibody (SMS+Ab) complex was measured as before. When compared to the rate of DAB polymerization by SMS alone, the SMS+Ab complex polymerizes DAB at a rate more than 5000 times that of SMS on a moles of miniSOG domain basis (SMS+Ab = 6.978 min^-1^ nmol^-1^, SMS = 9.761x10^-4^ min^-1^ nmol^-1^), indicating the presence of additional ROS (S4 Fig).

**Fig 3:**
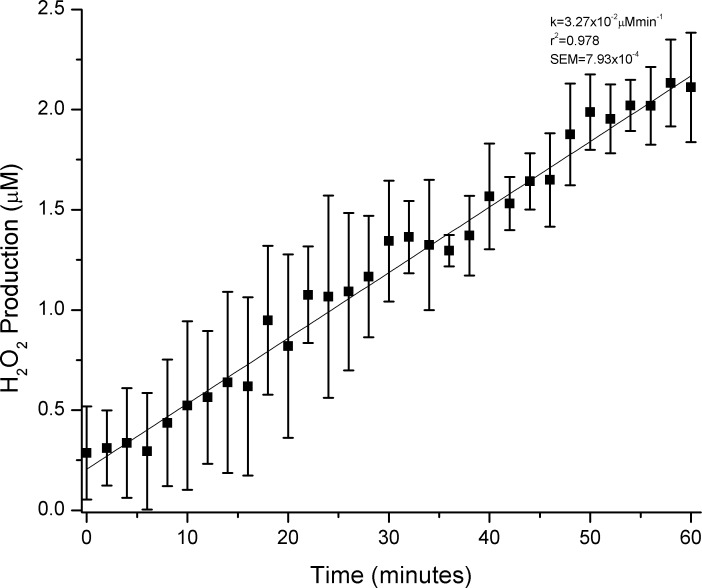
H_2_O_2_ Production of the SMS+EAb complex. H_2_O_2_ production by the SMS-antibody complex as determined by the Amplex Red method. The rate of H_2_O_2_ production was found to be 3.27x10^-2^ μM min^-1^ (r^2^ = 0.978, SEM = 7.93x10^-4^). Error bars represent the standard deviation of 5 samples.

Using the Amplex Red Method [50], the H_2_O_2_ production of the SMS+*E*. *coli* antibody (EAb) complex was investigated and was found to produce 2.11μM H_2_O_2_ after 1 hour of exposure to 450nm light (Fig 3), an amount comparable to that produced from TiO_2_ thin films [63]. The rate of H_2_O_2_ production was determined by subtracting the negligible H_2_O_2_ produced from either BSA+EAb or SMS+BSA mixtures and was found to be 3.27x10^-2^μM min^-1^. Previously, ambiguous ROS indicators have led to erroneous conclusions regarding specific ROS identifications; therefore, Amplex Red was chosen over other H_2_O_2_ indicators due to its specificity and low initial fluorescence of the substrate [50].

Previous studies using Amplex Red to detect H_2_O_2_ generation by antibodies have determined H_2_O_2_ generation rates to be 57nM min^-1^ or 0.06nmol H_2_O_2_ min^-1^ mg^-1^ protein [39]. The SMS+EAb complex produces H_2_O_2_ at a much higher rate per mg total protein present (21.8μmol H_2_O_2_ min^-1^ mg^-1^) than other antibody complexes. This increased H_2_O_2_ production rate may be due to the more efficient ^1^O_2_ production by SMS compared to other photosensitizers and/or the increased proximity of ^1^O_2_ generation to the antibody catalytic site due to streptavidin-biotin binding. Increased levels of H_2_O_2_ production compared to other antibody systems indicate that SMS+EAb should provide higher levels of disinfection than other antibody based methods.

**Fig 4:**
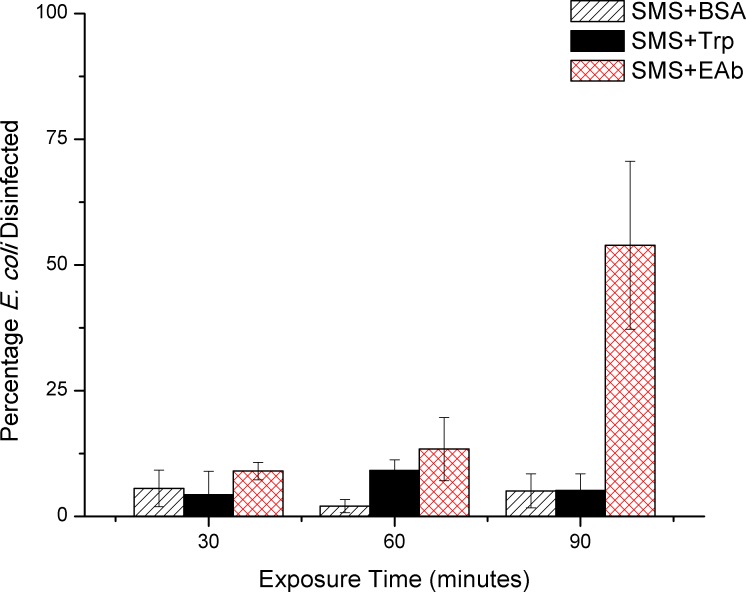
Disinfection of *E*. *coli* using SMS and Antibodies. Percentage of *E*. *coli* disinfected by SMS+BSA, SMS+Trp, and SMS+EAb as measured by the Baclight Live/Dead Bacterial Viability Kit. SMS and EAb were used at concentrations of 16.67μM and 25μM, respectively. Error bars represent the standard deviation of at least 3 samples.

**Fig 5:**
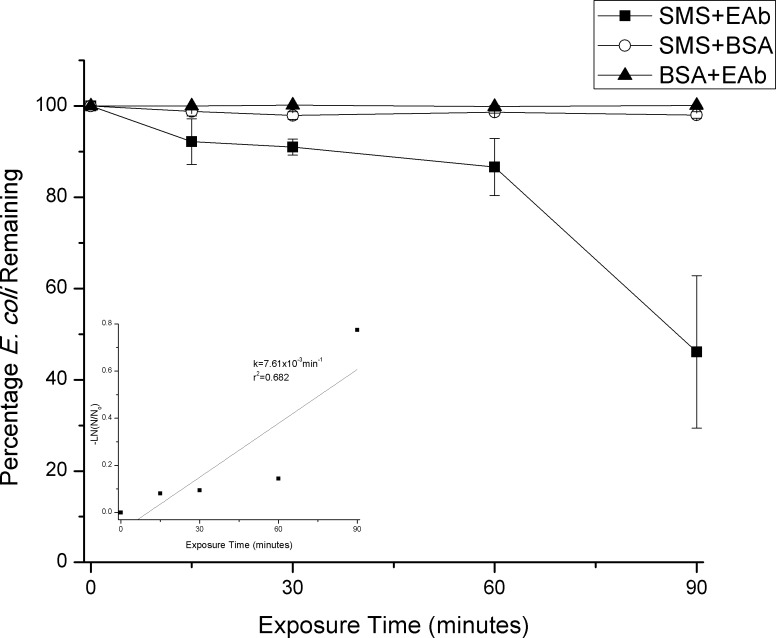
SMS+EAb Mediated Disinfection of *E*. *coli*. Percentage of *E*. *coli* disinfected by the SMS+EAb, SMS+BSA, and BSA+EAb mixtures as measured by the Baclight Live/Dead Bacterial Viability Kit. SMS and EAb were used at concentrations of 16.67μM and 25μM, respectively. Error bars represent the standard deviation of 3 samples. Inset shows data used to determine the rate constant, k, derived by fitting the natural log of (N/No) versus exposure time to Chick’s Law where N is the time dependent bacterial population and No is the starting population [51].

As expected, disinfection was much more efficient with 16.67μM SMS combined with 25μM of an *E*. *coli* specific antibody (EAb),with 53.9% of *E*. *coli* removed after 90 minutes of exposure (Fig 4). This result showed an improvement over other reported photocatalytic disinfection systems which were limited to 40% disinfection over the same time frame [58]. Disinfection as a result of light intensity, ^1^O_2_ produced by SMS, and antibody binding was determined using *E*. *coli* and controls were prepared, exposed and measured as described for previous disinfection experiments. Bacterial removal under control conditions was found to be minimal as the controls showed a maximum of 2% disinfection (Fig 5). The first-order kinetics, described by Chick's Law ([51]) were used to determine the disinfection rate constant for the SMS+EAb complex, which was 7.61x10^-3^min^-1^ (Fig 5, inset), higher than reported constants for Rose Bengal disinfection [64]. While it appears possible that our system departs from first order kinetics, the simplest model, described by Chick's Law, enables comparison to previously reported disinfection rates.

### Targeted Disinfection

**Fig 6:**
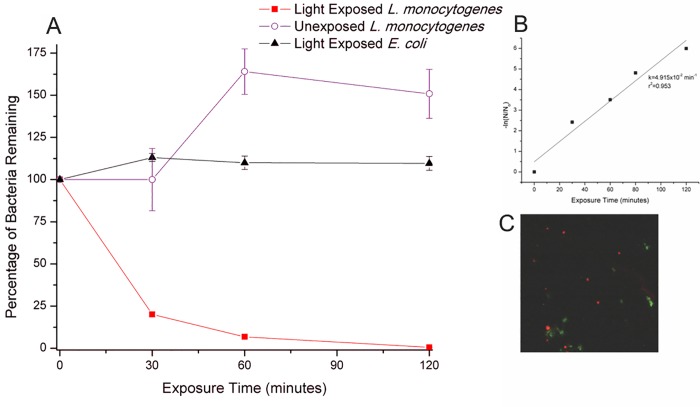
Targeted Disinfection of *L*. *monocytogenes*. A) Disinfection of light exposed *L*. *monocytogenes* in a mixed population with *E*. *coli*. SMS and LAb were used at 13.29nM and 66.44nM, respectively. Error bars represent the standard deviation of at least 3 trials. B) Graph of the natural log of (N/No) versus disinfectant exposure time fit to Chick’s Law for selective disinfection of *L*. *monocytogenes*, where N is time dependent bacterial population and No is the starting population. k, the Chick's Law constant, was found to be 4.915x10^-2^min^-1^ (r^2^ = 0.953). C) Fluorescent demonstration of selective labeling using the SMS+Ab system. *E*. *coli* is labeled with SMS+EAb (green) while *L*. *monocytogenes* is stained with Texas Red-wheat germ agglutinin (red). Magnification is 60X.

Following successful disinfection with *E*. *coli*, selective disinfection of *L*. *monocytogenes* in a mixed bacterial population of *L*. *monocytogenes* and *E*. *coli* was examined. *L*. *monocytogenes* was chosen as the target organism, since it is a gram positive bacteria (in contrast to *E*. *coli*) with well documented human pathogenicity, including recent food-borne outbreaks [65,66,67], where a biologically degradable disinfectant might be of value. After confirmation of H_2_O_2_ production by the *L*. *monocytogenes* antibody (LAb, S5 Fig), selective labeling of the *E*. *coli* and *L*. *monocytogenes* mixture was confirmed by fluorescent microscopy (Fig 6C). Imaging showed selective labeling of bacteria and no overlap in SMS+EAb or Texas Red-wheat germ agglutinin labeled bacteria. Following confirmation of appropriate antibody discrimination, selective disinfection was tested. The combination of SMS (13.29nM) and LAb (66.44nM) resulted in an 80% reduction in *L*. *monocytogenes* after 30 minutes and a 2-log reduction after 2 hours of exposure (Fig 6A). The dramatic population decrease in light exposed L. monocytogenes after 30 minutes can be correlated with the apparent H2O2 production lag, followed by a sharp increase (S5 and S6 Figs), resulting in improved disinfection. Exposure to 450nm light and the SMS+LAb complex were found to have a negligible effect on the *E*. *coli* present, indicating a selective disinfection of *L*. *monocytogenes* in the presence of the LAb (Fig 6A). Previously, ROS production from antibodies was reported to result in non-specific disinfection; however, these investigations were limited to monocultures exposed to a saturating concentration of antibody [43,45]. In the absence of light, the SMS+LAb complex was found to have little to no influence on the disinfection of *L*. *monocytogenes*, with some of the *L*. *monocytogenes* populations actually increasing in the dark (Fig 6A). First order kinetics based on Chick's Law of disinfection were used to determine the disinfection rate constant for the SMS+LAb complex, which was found to be 4.915x10^-2^min^-1^ (Fig 6B). Our disinfection results for *L*. *monocytogenes* follow the first order kinetics of Chick's Law more closely than the results for *E*. *coli*, perhaps due to the single bilayer membrane structure of *L*. *monocytogenes* (versus the multi layered lipid membrane of *E*. *coli*) which could lower the amount of oxidant required to impart fatal damage/membrane permeability.

Selective disinfection of *L*. *monocytogenes* using the SMS+LAb complex was found to be much more efficient than disinfection of *E*. *coli* using the SMS+EAb complex. The majority of the *L*. *monocytogenes* disinfection occurred within the first 30 minutes of exposure, with 80% *L*. *monocytogenes* removal, making the SMS+LAb complex much more efficient than the SMS+EAb complex which showed less than 10% inactivation after 30 minutes (Fig 6) and other photocatalysts used for disinfection that show less than 20% removal in the same time span [58]. This dramatic reduction in necessary contact time for our photocatalyst compared to other photosensitizers [58,68] could be due to the close proximity of the ROS generated by the SMS+LAb complex to the target cell membrane.

Continuous photocatalytic functionality was demonstrated under longer light irradiance which resulted in a 2-log reduction in *L*. *monocytogenes* after 2 hours. The first-order rate constant, for this system was found to be 4.915x10^-2^min^-1^, higher than reported constants for rose bengal disinfection [64] and more than six times higher than that of the SMS+EAb complex. It is known that *L*. *monocytogenes* transcribes SigB related genes specifically as a stress response to blue light [69], indicating that the bacteria are equipped with a defensive mechanism for the light exposure itself. The SMS+LAb rate constant value reflects both the previously documented increased susceptibility of Gram positive bacteria to photocatalytic inactivation via ROS [70,71,72] as well as the metabolic costs of SigB due to exposure to 450nm light.

Despite previous research indicating that antibody binding is not required for antibody based disinfection methods [43], our studies found it to be required for effective disinfection of *L*. *monocytogenes* at the levels of antibody used (66.44nM). The SMS+LAb complex demonstrated the ability to selectively remove *L*. *monocytogenes* from solutions containing a mixed population of *L*. *monocytogenes* and *E*. *coli*. This is in contrast to previous work demonstrating antibody disinfection as a nonspecific phenomenon [43]; however, the concentration of antibody used in those experiments is 80X higher than in this work, an amount that would certainly generate levels of H_2_O_2_ that would result in nonspecific disinfection.

## Conclusions

ROS mediated disinfection methods have been previously applied for use in a variety of situations for nonspecific disinfection of contaminated waters and surfaces. However, current disinfection systems present significant human and environmental toxicity concerns, rendering them undesirable for widespread use. Additionally, many of these mechanisms nonspecifically disinfect microorganisms, an effect that is often unnecessary.

The SMS-antibody based disinfection system presented here allows for amplification of ROS to efficiently remove bacteria from solution. While both system components produce ROS individually, only in combination do they consistently produce effective bacterial disinfection. Unlike other ROS disinfection mechanisms, the SMS-antibody complex is incapable of crossing the cell membrane due to size limitations. Further, the highly selective binding of antibodies to their target antigen ensures that the ROS are able to selectively oxidize the desired microorganism and not other system components. The ROS produced by free SMS or antibodies was found to be insufficient for effective bacterial inactivation which could indicate an extension of the system to be applied to targeted disinfection. Complete disinfection can be undesirable for some applications and general oxidation of all microorganisms is an ineffective strategy when only a small fraction of microorganisms are pathogens. The potential targeted and selective nature of the SMS-antibody system provides a method to specifically oxidize only biohazards of concern, making it ideal for applications where maintaining biodiversity is critical, such as the surface microbiome of immunocompromised individuals, and where pathogen outbreaks occur in food and water sources, since these must be removed while minimizing catalyst-derived hazards of consumption.

## Supporting Information

S1 Fig SMS Disinfection of *E*. *coli*. Percentage of *E*. *coli* disinfected by SMS exposed to 450nm light and SMS kept in the dark as measured by the Live/Dead Baclight Bacterial Viability Kit. Error bars represent the standard deviation of 4 samples.
(TIF)

S2 Fig Multi-SOG Disinfection of *E*. *coli*. Disinfection of *E*. *coli* using SMS, tandem miniSOG (tanSOG), and trimeric miniSOG (triSOG) as measured by the Live/Dead Baclight Bacterial Viability Kit. Error bars represent the standard deviation of 4 samples.
(TIF)

S3 Fig Tryptophan Assisted Disinfection of *E*. *coli*. A) Percentage of *E*. *coli* disinfected using SMS, SMS+Trp or SMS+PEG as measured by the Live/Dead Baclight Bacterial Viability Kit. Error bars represent the standard deviation of at least 3 samples. B) Percentage of *E*. *coli* disinfected using varying concentrations of tanSOG with Trp as measured by the Live/Dead Baclight Bacterial Viability Kit. Error bars represent the standard deviation of 3 samples.
(TIF)

S4 Fig SMS and Antibody Catalyzed DAB Polymerization. DAB polymerization, tracked by absorbance at 595nm,was used to measure the rates of ROS generation by SMS+Ab and BSA+Ab. All values represent the average of three separate measurements.
(TIF)

S5 Fig H_2_O_2_ Production of SMS and the *L*. *monocytogenes* Antibody. H_2_O_2_ production of the SMS+LAb complex as measured by the Amplex Red method. The linear rate of H_2_O_2_ production was found to be 3.958x10^-1^μM min^-1^, a rate that is the product of subtracting the negligible H_2_O_2_ produced by antibody with the non-light harvesting protein bovine serum albumin (BSA) (or equivalently negligible SMS and BSA) from the antibody with SMS. Error bars represent the standard deviation of 4 samples.
(TIF)

S6 Fig Sigmoidal curve fitting of the H_2_O_2_ Production of SMS and the *L*. *monocytogenes* antibody. Rate determination of H_2_O_2_ production of the SMS+LAb complex using a Boltzmann sigmoidal curve yields a time constant (dx) value of 8.482 (SEM = 0.352) and a rate (1/dx) of 1.179x10^-1^ min^-1^. Error bars represent the standard deviation of 4 samples.
(TIF)

S1 File Additional Methods and Data Discussion. (DOCX)
