# Supplementary figures and images for: Selective Photocatalytic Disinfection by Coupling StrepMiniSog to the Antibody Catalyzed Water Oxidation Pathway
Source: PLoS One. 2016 Sep 12;11(9):e0162577. doi: 10.1371/journal.pone.0162577 (PMC5019378; doi:10.1371/journal.pone.0162577)

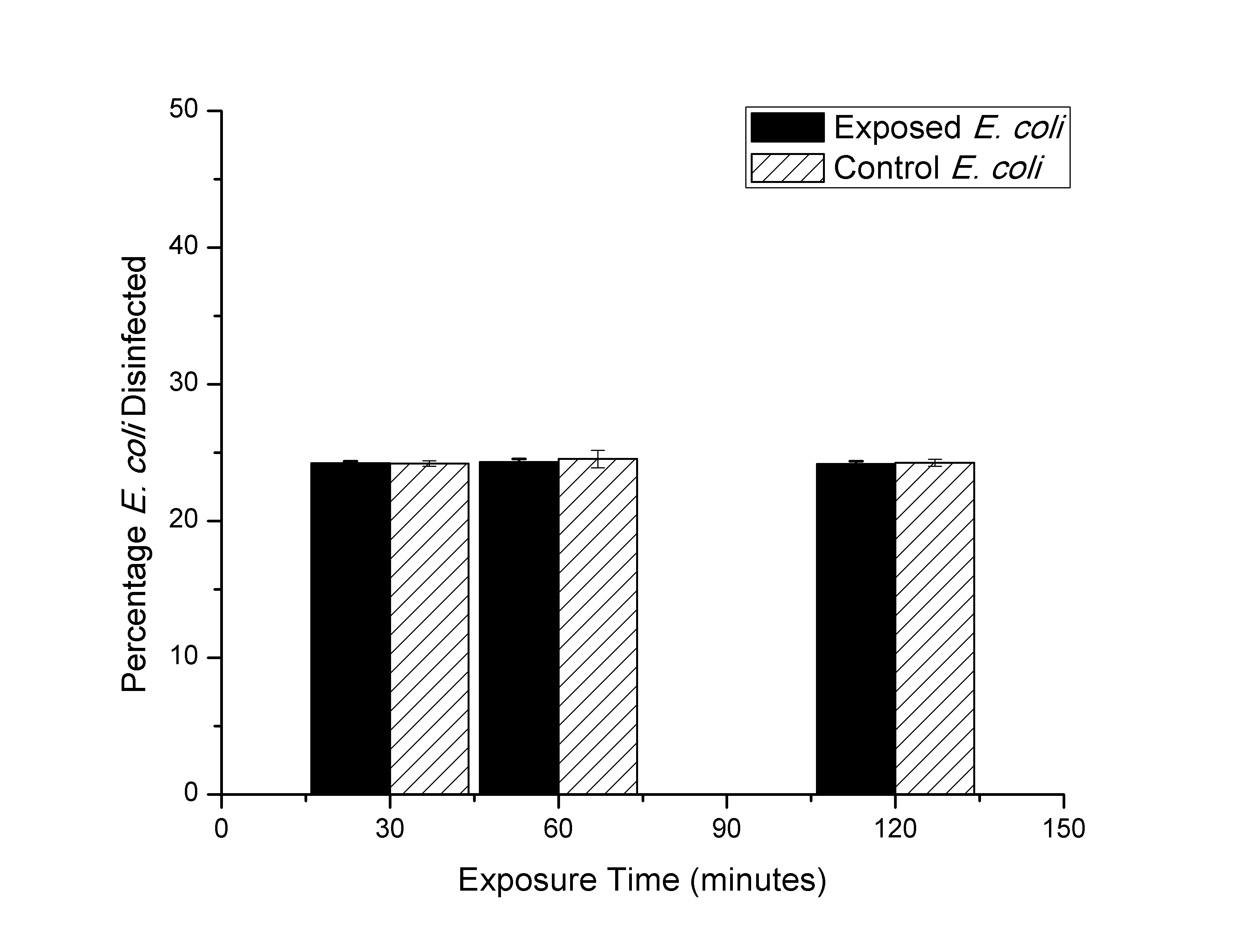

Supplement: S1 Fig — Percentage of E. coli disinfected by SMS exposed to 450nm light and SMS kept in the dark as measured by the Live/Dead Baclight Bacterial Viability Kit. Error bars represent the standard deviation of 4 samples. (TIF) [file pone.0162577.s001.tif]

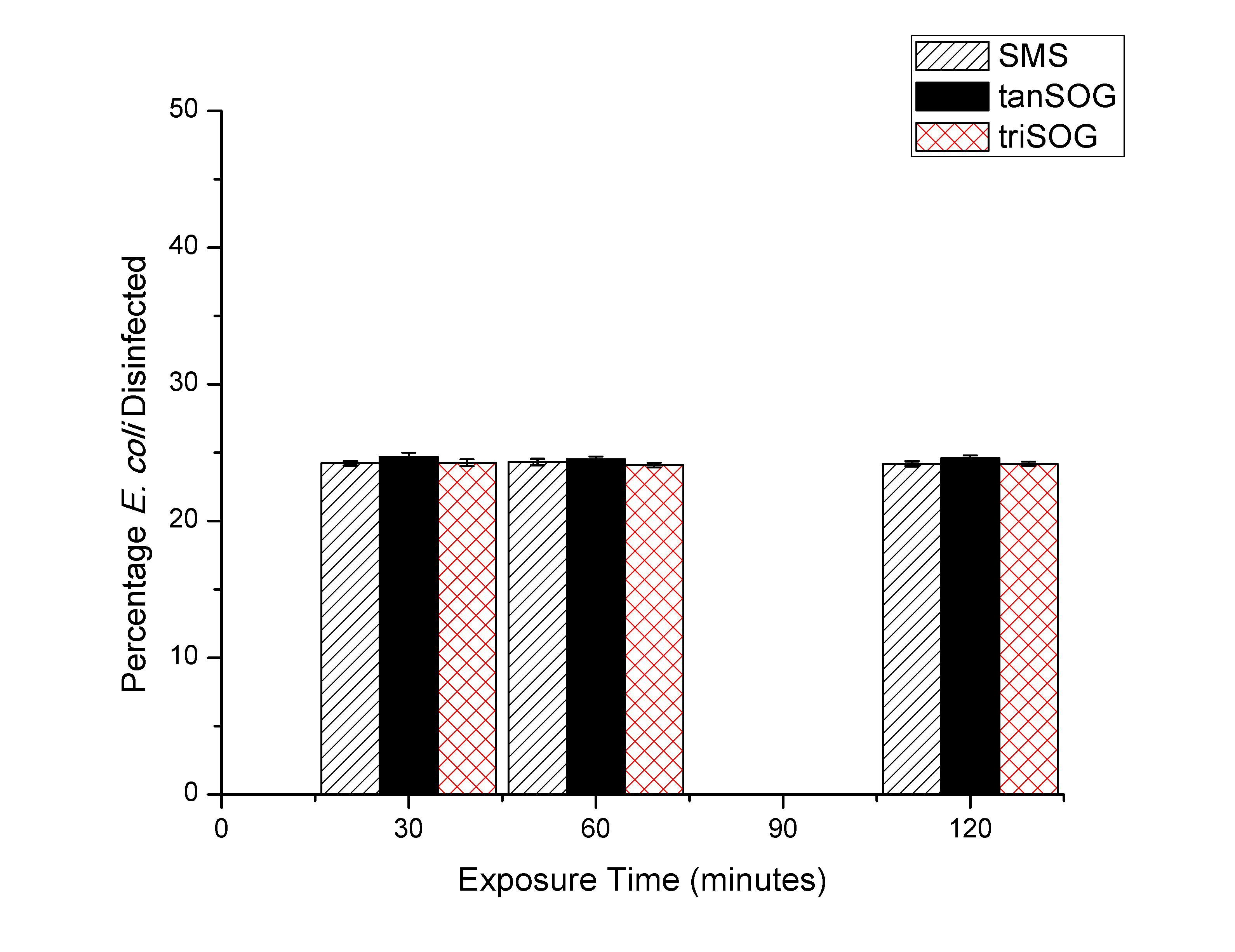

Supplement: S2 Fig — Disinfection of E. coli using SMS, tandem miniSOG (tanSOG), and trimeric miniSOG (triSOG) as measured by the Live/Dead Baclight Bacterial Viability Kit. Error bars represent the standard deviation of 4 samples. (TIF) [file pone.0162577.s002.tif]

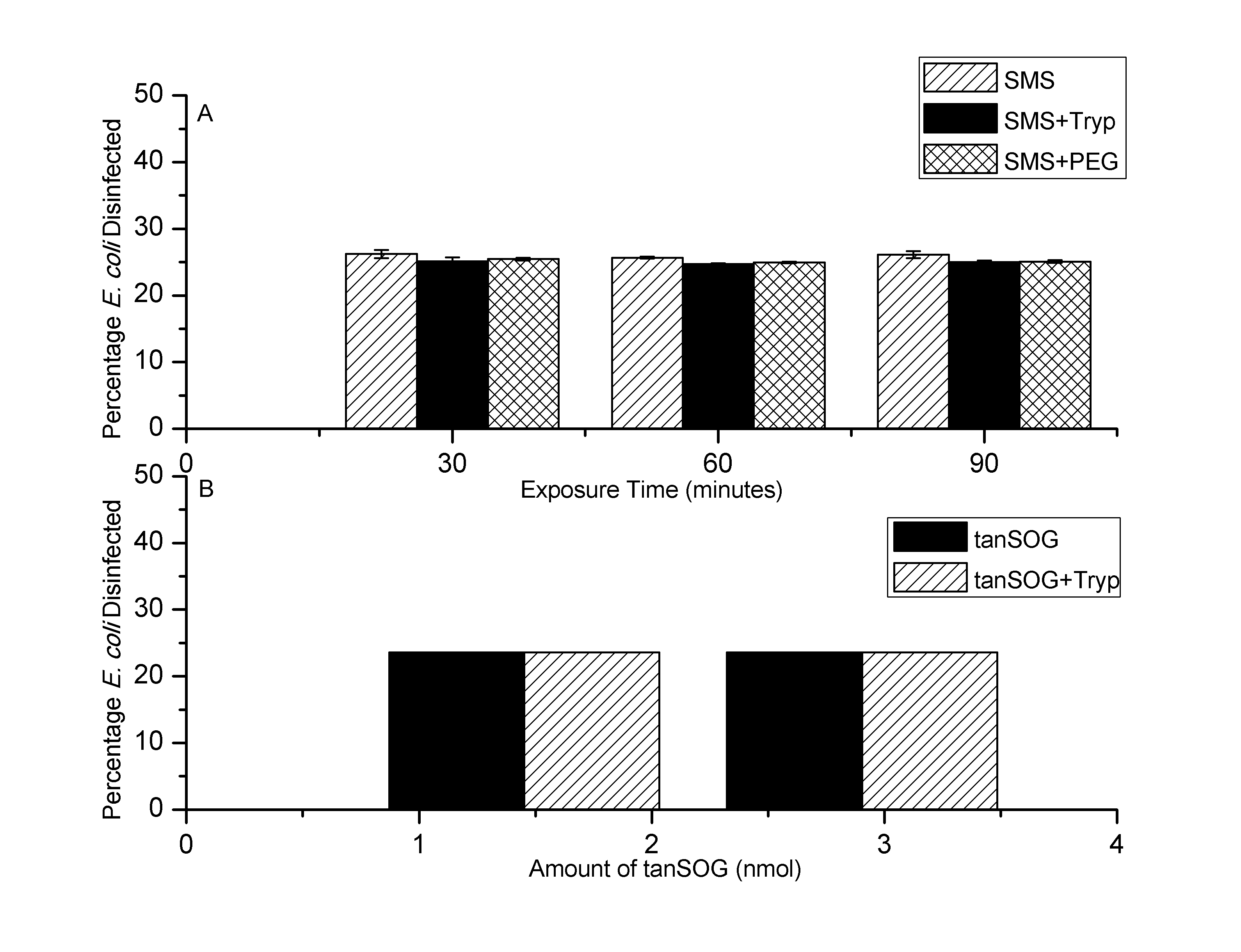

Supplement: S3 Fig — A) Percentage of E. coli disinfected using SMS, SMS+Trp or SMS+PEG as measured by the Live/Dead Baclight Bacterial Viability Kit. Error bars represent the standard deviation of at least 3 samples. B) Percentage of E. coli disinfected using varying concentrations of tanSOG with Trp as measured by the Live/Dead Baclight Bacterial Viability Kit. Error bars represent the standard deviation of 3 samples. (TIF) [file pone.0162577.s003.tif]

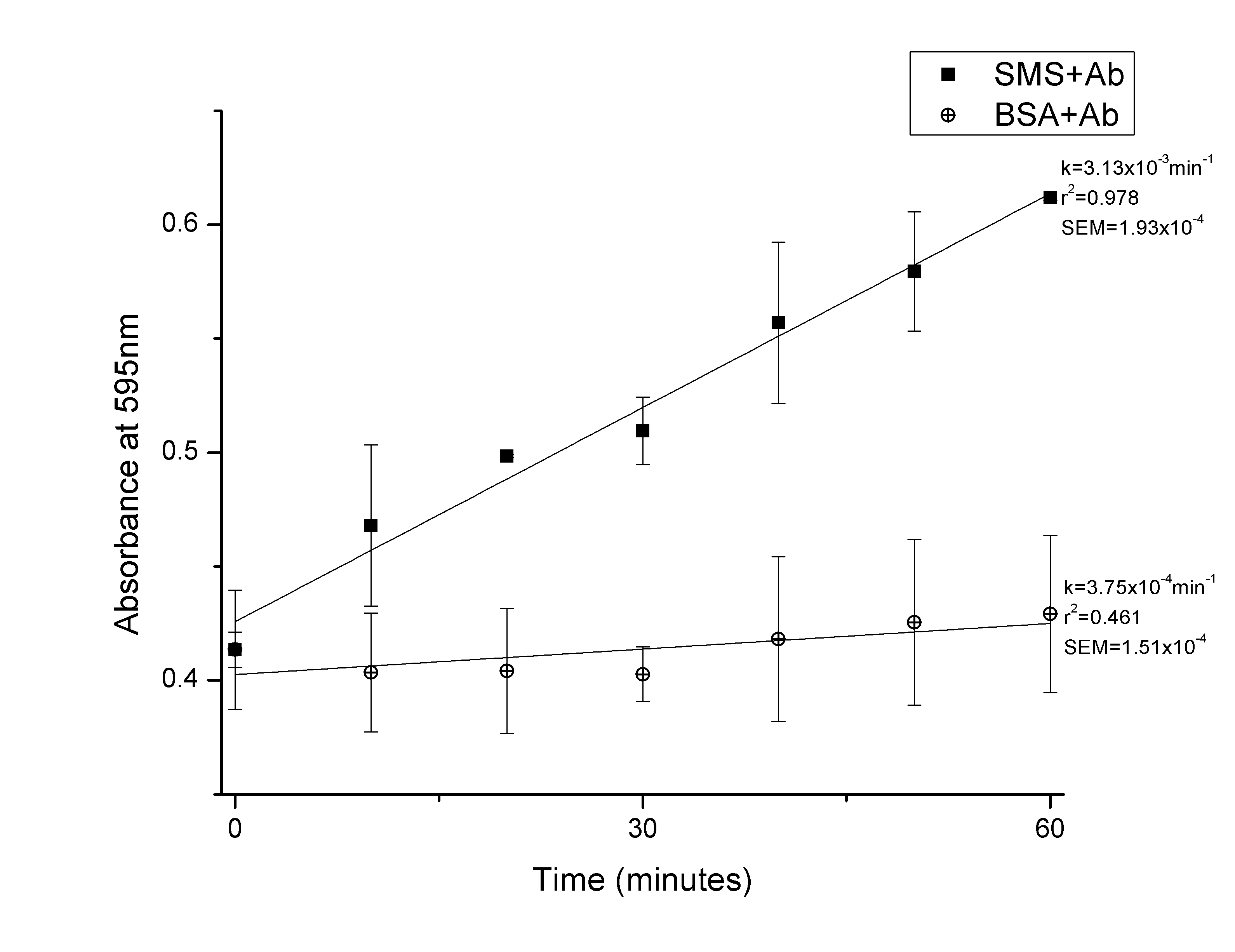

Supplement: S4 Fig — DAB polymerization, tracked by absorbance at 595nm,was used to measure the rates of ROS generation by SMS+Ab and BSA+Ab. All values represent the average of three separate measurements. (TIF) [file pone.0162577.s004.tif]

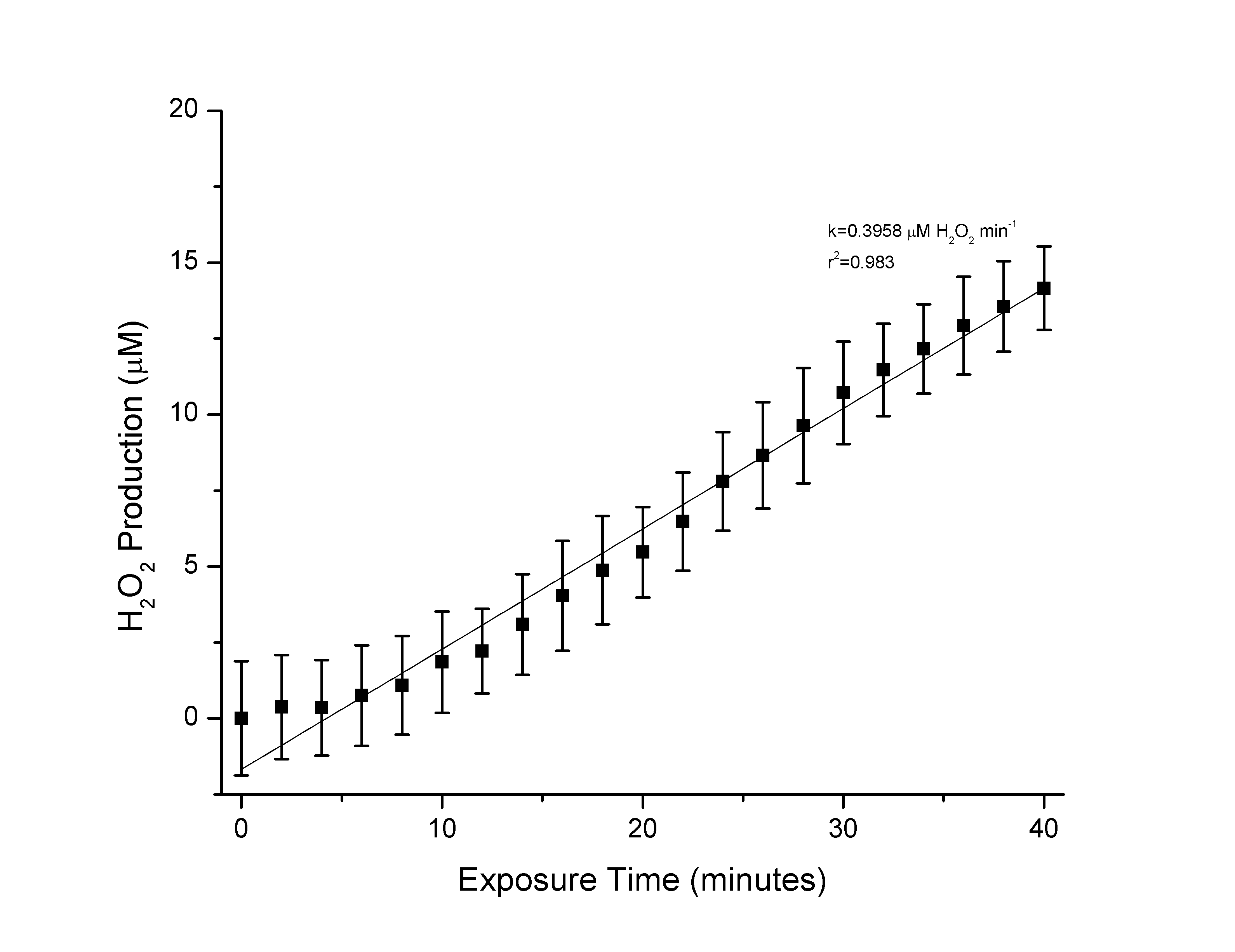

Supplement: S5 Fig — H2O2 production of the SMS+LAb complex as measured by the Amplex Red method. The linear rate of H2O2 production was found to be 3.958x10^-1^μM min-1, a rate that is the product of subtracting the negligible H2O2 produced by antibody with the non-light harvesting protein bovine serum albumin (BSA) (or equivalently negligible SMS and BSA) from the antibody with SMS. Error bars represent the standard deviation of 4 samples. (TIF) [file pone.0162577.s005.tif]

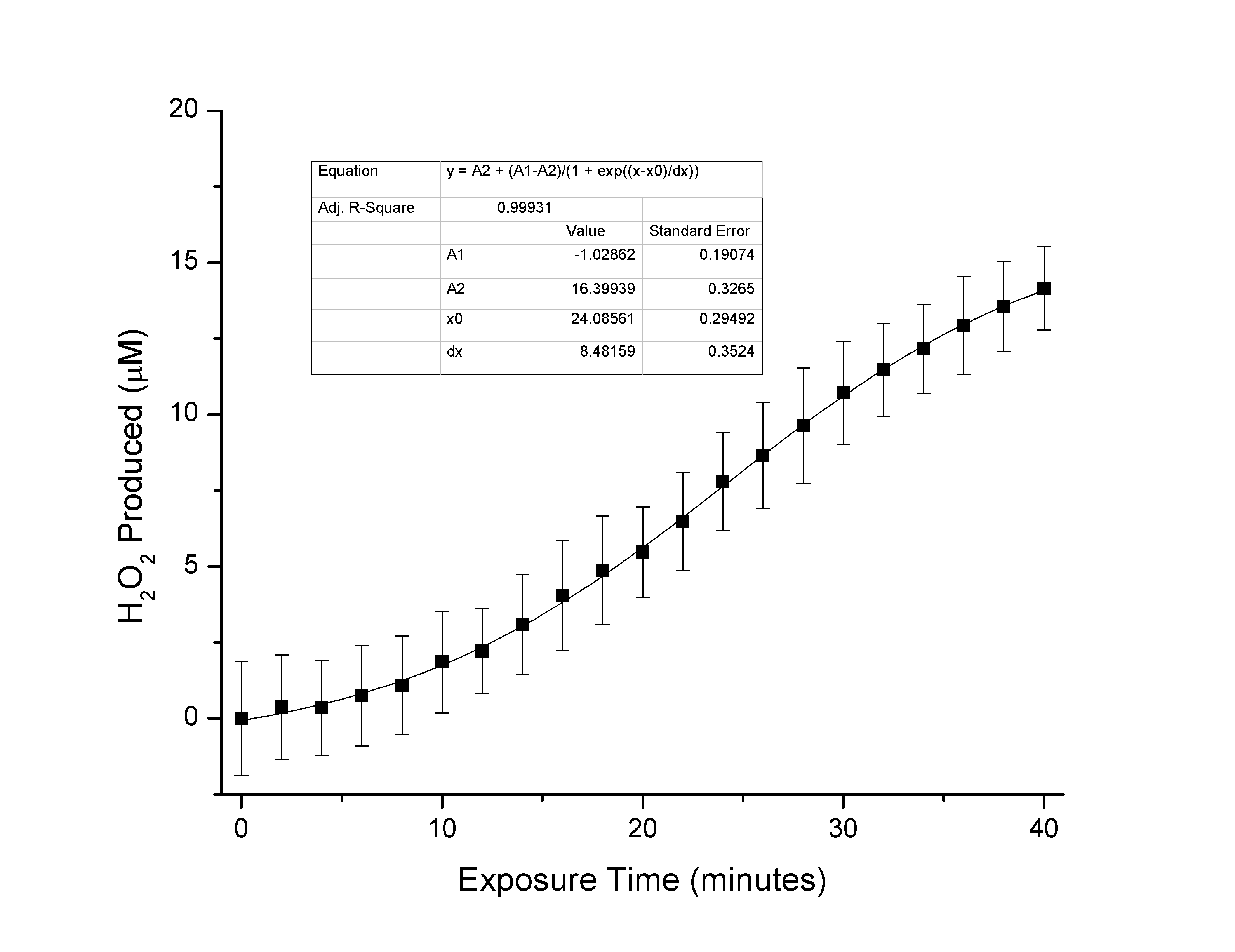

Supplement: S6 Fig — Rate determination of H2O2 production of the SMS+LAb complex using a Boltzmann sigmoidal curve yields a time constant (dx) value of 8.482 (SEM = 0.352) and a rate (1/dx) of 1.179x10-1 min-1. Error bars represent the standard deviation of 4 samples. (TIF) [file pone.0162577.s006.tif]
