# Supplementary material for: Selective Photocatalytic Disinfection by Coupling StrepMiniSog to the Antibody Catalyzed Water Oxidation Pathway
Source: PLoS One. 2016 Sep 12;11(9):e0162577. doi: 10.1371/journal.pone.0162577 (PMC5019378; doi:10.1371/journal.pone.0162577)
Supplement: S1 File — (DOCX) [file pone.0162577.s007.docx]

**Disinfection with SMS**

Surprisingly, disinfection studies using only SMS showed minimal disinfection efficiency, indicating that ^1^O_2_, as it is generated from SOG is not effective at killing bacteria (S1 Fig).

**S1 Fig. SMS Disinfection of *E. coli*.** Percentage of *E. coli* disinfected by SMS exposed to 450nm light and SMS kept in the dark as measured by the Live/Dead Baclight Bacterial Viability Kit. Error bars represent the standard deviation of 4 samples.

**Disinfection with tanSOG and triSOG**

Following the inability of SMS to effectively photodisinfect *E. coli*, the photodisinfection capabilities of tanSOG and triSOG were examined. Although these proteins produce ROS at a lower rate than SMS [[1](#_ENREF_1)], the smaller protein size could lead to faster diffusion kinetics, potentially increasing the rate of disinfection. Disinfection experiments were carried out using purified tanSOG and triSOG and *E. coli* at a density of 10^6^cells/mL. Protein was added in amounts to ensure identical number of miniSOGs in each sample. Following irradiation with 450nm light (460 µmol m^-2^ s^-1^), excess protein was removed from solution and cells were stained using the Live/Dead Baclight Bacterial Viability Kit. Fluorescent measurements were taken in the FlexStation3 using the wavelengths previously described.

Use of tanSOG and triSOG in disinfection did not appear to improve removal efficiency as all disinfection percentages remained under 25% (S2 Fig). Disinfection studies using the created miniSOG proteins showed minimal disinfection efficiency, indicating that ^1^O_2_, as it is generated from extracellular miniSOG, is not effective at killing bacteria.

**S2 Fig. Multi-SOG Disinfection of *E. coli*.**  Disinfection of *E. coli* using SMS, tanSOG, and triSOG as measured by the Live/Dead Baclight Bacterial ViabilityKit. Error bars represent the standard deviation of 4 samples.

**Longer Lived ROS**

Following the ineffective results with only ^1^O_2_ generating proteins, a longer lived ROS was sought to improve disinfection. Previous work has suggested the formation of O_3_ from certain amino acids, including tryptophan, in the presence of ^1^O_2_ that led to removal of *E. coli* from solution [[2](#_ENREF_2)], and also the reported IgA catalyzed formation of O_3_ from ^1^O_2_ [[3](#_ENREF_3)]. Further, tryptophan residues present in antibodies have been identified as the catalytic site for H_2_O_2_ production [[4](#_ENREF_4)], suggesting a protein mediated pathway for a longer lived ROS from ^1^O_2_ generation. To facilitate production of a longer lived ROS, tryptophan was added to disinfection trials using SMS and tanSOG (S3 Fig). Disinfection trials and subsequent quantification were carried out as done in previous disinfection experiments with tryptophan added to a final concentration of 13.5µM. Polyethylene glycol (PEG) was added in equimolar amounts to some replicates to serve as an osmotic control. For both SMS disinfection experiments, addition of either 13.5µM tryptophan or 13.5µM PEG was found using ANOVA to significantly *decrease* the amount of disinfection (p<0.05, S3A Fig). Using 13.5µM tryptophan in disinfection experiments with tanSOG was found to have no statistically significant impact using ANOVA analysis on disinfection rates (p>0.05, S3B Fig). However, use of ANOVA determined that the increase in SOG concentration was found to significantly increase the levels of *E. coli* disinfection seen for tanSOG (p<0.05, S3B Fig).

**S3 Fig. Tryptophan Assisted Disinfection of *E. coli*.** A) Percentage of *E. coli* disinfected using SMS, SMS+Tryp or SMS+PEG as measured by the Live/Dead Baclight Bacterial Viability Kit. Error bars represent the standard deviation of at least 3 samples. B) Percentage of *E. coli* disinfected using varying concentrations of tanSOG with Tryp as measured by the Live/Dead Baclight Bacterial Viability Kit. Error bars represent the standard deviation of 3 samples.

**SMS+Ab Catalyzed DAB Polymerization**

Combining an antibody with SMS should result in increased ROS production (H_2_O_2_) upon blue light irradiation. Therefore, general ROS production of the combined SMS and antibody was measured via DAB polymerization to confirm creation of a longer lived ROS by the ACWOP.

The SMS+Ab complex polymerized DAB at a rate of 2.032x10^-3^ min^-1^ (r^2^=0.927), 5X greater than the polymerization seen by BSA+Ab, indicating that the irradiated antibody alone produces minimal ROS when exposed to 450nm light (S4 Fig). When compared to the rate of DAB polymerization by SMS alone, the SMS+Ab complex polymerizes DAB at a rate more than 5000 times that of SMS on a moles of miniSOG domain basis (SMS+Ab=6.978 min^-1^ nmol^-1^, SMS=9.761x10^-4^ min^-1^ nmol^-1^), indicating the presence of additional ROS.

**S4 Fig. SMS and Antibody Catalyzed DAB Polymerization.** DAB polymerization, tracked by absorbance at 595nm,was used to measure the rates of ROS generation by SMS+Ab and BSA+Ab. All values represent the average of three separate measurements.

**H_2_O_2_ Production of SMS+LAb**

Due to reported variation in H_2_O_2_ production yields between different antibodies [[5](#_ENREF_5)], possibly attributed to variable total tryptophan content [[6](#_ENREF_6)], the H_2_O_2_ generating capabilities of the biotinylated *Listeria monocytogenes* antibody (LAb, Thermo Scientific) were also quantified using the Amplex Red method [[7](#_ENREF_7)]. Briefly, SMS and LAb (SMS+LAb) were mixed in a 1:4 molar ratio and added to the Amplex Red reaction solution. Controls were performed using BSA in the place of SMS and the LAb (BSA+LAb and SMS+BSA, respectively). Samples were exposed to 450nm light (460 µmol m^-2^ s^-1^) and fluorescence was measured every two minutes for 40 minutes using an excitation and emission of 568nm and 570nm, respectively, with excitation cutoff set at 570nm. Fluorescent measurements were taken in the FlexStation3 Plate Reader. All measurements were done in quadruplicate. Prior to use, sodium azide was selectively removed from the buffer via dialysis using 20KMW Slide-a-lyzer cartridges (Pierce), since this may interfere with ROS generation.

The linear H_2_O_2_ production rate of the SMS+LAb complex was found to be 0.3958µM min^-1^ (r^2^=0.983) using Amplex Red (S5 Fig), a rate over 10 times higher than that of the SMS+EAb complex, which was also found to be a statistically significant increase using ANOVA analysis (p<0.05). The SMS+LAb complex produced 14.16µM H_2_O_2_ after 40 minutes of exposure to 450nm light. Given this increased H_2_O_2_ generation rate of the SMS+LAb complex over the EAb, it is expected that disinfection using the LAb will show improved efficiency. A linear fit was chosen to provide easy comparison to both the H_2_O_2_ generation rate of the EAb used in this study, as well as previously reported values. While a Boltzmann sigmoidal curve with an improved fit (r^2^=0.999) provides a time constant (dx) value of 8.482 (SEM=0.352), it cannot be directly compared to the linear rate constant found for the EAb (S6 Fig).

Figure S5. H_2_O_2_ production of the SMS+LAb complex as measured by the Amplex Red method. The rate of H_2_O_2_ production was found to be 3.958x10^-1^µM min^-1^, a rate that is the product of subtracting the negligible H_2_O_2_ produced by antibody with the non-light harvesting protein bovine serum albumin (BSA) (or equivalently negligible SMS and BSA) from the antibody with SMS. Error bars represent the standard deviation of 4 samples.

**S5 Fig. H_2_O_2_ Production of SMS and the *L. monocytogenes* Antibody.** H_2_O_2_ production of the SMS+LAb complex as measured by the Amplex Red method. The rate of H_2_O_2_ production was found to be 3.958x10^-1^µM min^-1^, a rate that is the product of subtracting the negligible H_2_O_2_ produced by antibody with the non-light harvesting protein bovine serum albumin (BSA) (or equivalently negligible SMS and BSA) from the antibody with SMS. Error bars represent the standard deviation of 4 samples.

An improved fit for the LAb H_2_O_2_ production was found using a Boltzmann sigmoidal curve (r^2^=0.999, S6 Fig), providing a time constant (dx) value of 8.482 (SEM=0.352) and a rate (1/dx) of 1.179x10^-1^min^-1^.

**S6 Fig. Sigmoidal curve fitting of the H_2_O_2_ Production of SMS and the *L. monocytogenes* antibody.** Rate determination of H_2_O_2_ production of the SMS+LAb complex using a Boltzmann sigmoidal curve. Error bars represent the standard deviation of 4 samples.

**References**

1. Wurtzler EM, Wendell D. A Streptavidin-SOG Chimera for All-Optical Immunoassays. Biomacromolecules. 2013.

2. Yamashita K, Miyoshi T, Arai T, Endo N, Itoh H, et al. Ozone production by amino acids contributes to killing of bacteria. Proceedings of the National Academy of Sciences. 2008; 105: 16912-16917.

3. Uehara Y, Agematsu K, Kikuchi K, Matsuzaki S, Imai S, et al. Secretory IgA, Salivary Peroxidase, and Catalase-Mediated Microbicidal Activity during Hydrogen Peroxide Catabolism in Viridans Streptococci: Pathogen Coaggregation. Journal of Infectious Diseases. 2006; 194: 98-107.

4. Zhu X, Wentworth P, Wentworth AD, Eschenmoser A, Lerner RA, et al. Probing the antibody-catalyzed water-oxidation pathway at atomic resolution. Proceedings of the National Academy of Sciences of the United States of America. 2004; 101: 2247-2252.

5. Wentworth AD, Jones LH, Wentworth P, Janda KD, Lerner RA. Antibodies have the intrinsic capacity to destroy antigens. Proceedings of the National Academy of Sciences. 2000; 97: 10930-10935.

6. Wentworth P, Jones LH, Wentworth AD, Zhu X, Larsen NA, et al. Antibody catalysis of the oxidation of water. Science. 2001; 293: 1806-1811.

7. Zhou M, Diwu Z, Panchuk-Voloshina N, Haugland RP. A Stable Nonfluorescent Derivative of Resorufin for the Fluorometric Determination of Trace Hydrogen Peroxide: Applications in Detecting the Activity of Phagocyte NADPH Oxidase and Other Oxidases. Analytical Biochemistry. 1997; 253: 162-168.
